# Enhancing organizational processes for service innovation: strategic organizational counseling and organizational network analysis

**DOI:** 10.3389/frma.2024.1270501

**Published:** 2024-01-29

**Authors:** Silvia Marocco, Mara Marini, Alessandra Talamo

**Affiliations:** Department of Social and Developmental Psychology, Sapienza University of Rome, Rome, Italy

**Keywords:** service innovation, organizational processes, Organizational Network Analysis, Strategic Organizational Counseling, methods

## Abstract

Previous studies have primarily focused on product innovation, overlooking the examination of organizational processes. This limited perspective poses a theoretical and practical gap as it primarily considers the external aspects of innovation. On the contrary, organizational processes play a crucial role in improving and creating internal operations necessary for product/service innovation success. To this aim, this paper presents a novel approach to enhancing service innovation within complex organizations by integrating Strategic Organizational Counseling (SOC) and Organizational Network Analysis (ONA) methodologies. More specifically, SOC supports organizations in understanding and defining the professional families that need to be triggered in the service ideation, delivery and commercialization process, especially in the case of complex organizations with multiple departments. Secondly, ONA enables the identification of the intra-organizational nodes within the professional families that, due to their social position and other personal characteristics, can be actively engaged as Ambassadors for the promotion of innovation practices. By focusing on intra-organizational processes, understanding role-related needs, and selecting influential organizational actors, this approach provides a new perspective on the service innovation process, assuming both a micro and macro viewpoint. The paper also highlights the importance of cyclically monitoring the proposed workflow to adapt to the dynamic nature of innovation.

## Introduction

The issue of innovation is not recent in the literature, and numerous scholars have defined it in a variety of complex and different ways, focusing on various dimensions and outcomes of innovation (i.e., products) or on the specificities of enterprises leading to innovative products (Liao et al., 2007; Kafetzopoulos and Psomas, 2015). In particular, previous research has focused mainly on *product innovation*, neglecting *organizational process innovation* (Piening and Salge, 2015). Indeed, while much has been written about user-centered design for the development of innovative products and services (Brown, 2009; Stickdorn and Schneider, 2011; Talamo et al., 2011, 2021), still more has to be done to assist organizations in responding to the complexity that characterizes innovation processes (Anderson, 1999). This *product-oriented* approach is problematic as it only considers the most visible aspect of innovation, the one connected to the external market. Another level of complexity in this field derives from the contemporary shift from *product-centricity* to *service-centricity*, which has witnessed a pervasive transformation in the economic and productive battleground (Pine and Gilmore, 1999). The intangible nature of services, indeed, poses a challenge in effectively communicating the service's valuable performance to external audiences beyond the provider organization. This, in turn, requires a high level of cross-functional cooperation and knowledge exchange among various organizational members at multiple levels.

To address these challenges and ensure successful innovation, the S*ectoral Innovation Systems Framework* (Malerba, 2002) adopts a multidimensional, integrated and dynamic approach in which intra and inter-organizational interactions are fundamental to respond effectively to the complexity of contemporary organizational systems (e.g., Stacey, 1995).

In this perspective, innovation also becomes a complex phenomenon that involves internal and external recombination processes. Consequently, an exclusive emphasis on the external side of innovation, as has been done so far (Piening and Salge, 2015), overlooks a crucial aspect required for leading service innovation toward success: the innovation of internal organizational processes, essential for preparing to engage with external interactions and ensuring efficient service delivery. In fact, companies should be supported in managing internal processes in the different phases, from *Ideation*, to *Project Selection, Product Development*, until *Commercialization* (Jaruzelski et al., 2005). This approach not only helps prevent collaboration failures that can significantly impede the success of the innovation process (Jaruzelski et al., 2005; Nambisan and Sawhney, 2011) but also enables organizations to gain insights into their internal constraints and identify the resources needed to effectively achieve their innovation goals.

For this reason, in this contribution, we intend to underline the importance of internal organizational processes to effectively enhance service innovation in complex organizations, where complex means “*rich in structure”* (Sammut-Bonnici, 2015). Specifically, we propose integrating two applied research tools and methods to support organizations in creating a process to provide innovative services adequately. On the one hand, we introduce *Strategic Organizational Counseling* (SOC), an organizational consultancy methodology aimed at identifying and facilitating the intra-organizational processes that guide the service design toward its integration with the external market (Talamo et al., 2021). On the other hand, we incorporate *Organizational Network Analysis* (ONA) (e.g., Borgatti and Molina, 2003; Garcia, 2015), a sociometric approach designed and applied in organizational contexts to surface informal organizational networks and identify specific actors who can drive the service-life flow to next-level results. In this paper, we propose combining these research tools as the initial step organizations should take to establish the groundwork for effectively managing the complexity of innovation. As illustrated in the following paragraphs, in fact, SOC and ONA can provide organizations with helpful information and data to produce new knowledge and increase organizations' awareness of the internal needs required to address the complexity of innovative processes.

## Strategic Organizational Counseling (SOC): innovation starts from inner organizational processes

SOC is a methodology developed by the IDEaCT Social Lab of Sapienza that supports organizations in the development of services and in facilitating the organizational processes essential for the organization's success (Talamo et al., 2021). SOC methodology focuses on enhancing awareness on the implicit decision-making processes that underlie the development of innovative services in all phases of internal management. SOC allows for a deeper understanding of strategies that support the effectiveness of service delivery by looking at the different phases as a whole process, where each professional family plays a role in supporting the innovation delivery.

SOC uses dialogic sessions and maieutic narrative interview techniques to make different organizational actors become aware of how organizational structure and processes can be crucial in meeting the demands of potential customers. These sessions are goal-oriented interviews aimed at refining the flow of organizational processes, starting from service design and ending with external market integration. Hence, the intent of SOC is defining a flow of organizational processes–favorable and unfavorable–of each crucial professional family that may interrupt or facilitate the service delivery. During SOC, psychologists experienced in organizational counseling help participants elicit representations that guide the service delivery process at the organizational level. When several actors participate in these sessions, the alignment of representations by each of them is discussed and modeled together. According to the maieutic method, the participants receive reflective interventions guiding them toward constructing and eliciting the flow of organizational processes.

SOC sessions include three phases based on the following major themes:

**Phase 1:** *Mapping the professional families involved in the service delivery process and the relationships between them*. At this stage, the aim is to identify which professional families are involved and how they interact and communicate at each stage of innovation implementation (from ideation to delivery);
**Phase 2:** *Identifying specific roles of different professional families and their role-related needs focusing on service delivery*. At this stage, the aim is to understand the specific tasks of each professional family related to the service delivery process and detect their particular needs and challenges faced to perform their tasks effectively;
**Phase 3:** *Defining facilitating interventions to promote innovation of practices*. At this stage, the aim is to comprehend how specific practices of each professional family should be modified, reshaped or better supported through facilitating interventions to make the whole process more efficient.

**Figure 1:**
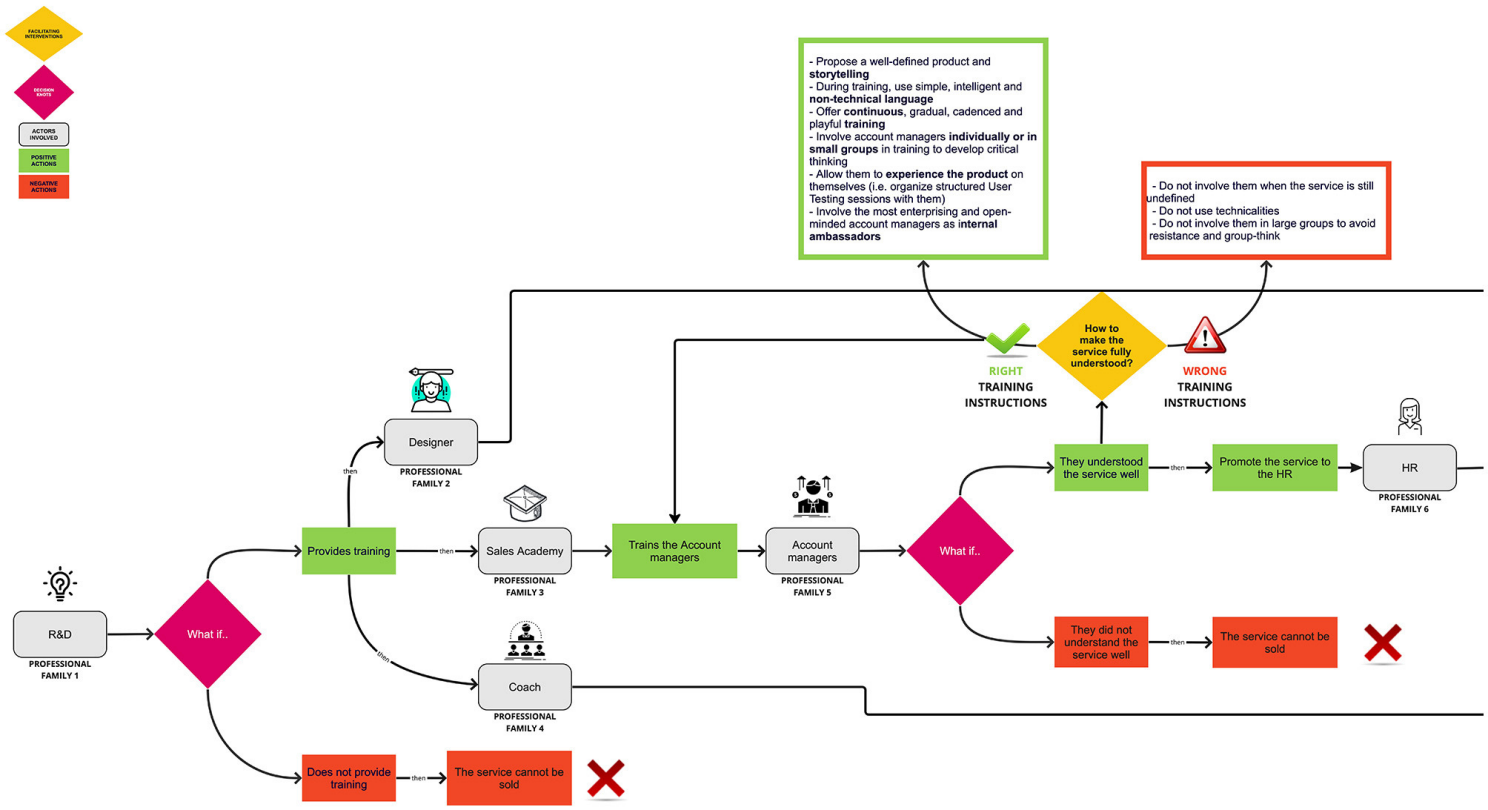
An example of organizational flow.

The result of these sessions is from time to time discussed and reworked by the participants themselves in a visual format (Figure 1) that is consolidated in the last meeting. This visual flow shows how the organizational processes, linked to various professional families, need to be governed to ensure the service's success. In particular, some *decision knots* emerge from the SOC sessions related to the professional families identified in the flow; each decision is connected to what happened before and enables what happens afterwards. These points are highlighted in the diagram as *fuchsia diamonds* and stand for “*what if”* questions. From there, two possible paths branch off:

- a positive one (in green), in which a favorable behavior of the professional family is made explicit, which allows it to continue with the life flow of the service;
- a negative one (in red), in which an unfavorable professional family's behavior interrupts the service life-flow.

Having a visual tool makes it easier for professional families to understand their role in empowering the whole process and the reason why innovation in organizational practices is needed. Below is an example to concretely understand the structure of the flow diagram (Figure 1).

## Organizational Network Analysis (ONA): innovation as a social product

In organizational research, there is a growing interest in Organizational Network Analysis (ONA; e.g., Borgatti and Molina, 2003; Cross et al., 2013; Garcia, 2015), a set of tools and methods for mapping and analyzing organizational networks (Cross et al., 2002; Ujwary-Gil, 2020). According to Wasserman and Faust (1994), connections among organizational members or units are crucial organizational resources because they represent flows of tangible and intangible assets. Through the use of ONA, in fact, organizations can integrate their formal structure with relational networks (informal structure) and make better use of their human resources (Michalski and Kazienko, 2014; Garcia, 2015; Ujwary-Gil, 2020). Specifically, by comparing formal and informal structures, companies can identify individuals able to facilitate their practices and, at the same time, become aware of the social relationships that promote/hinder the efficiency of organizational processes. In addition, comparing the visible and concealed organizational structures enables the identification of discrepancies between formal and informal roles in the organizational chart, allowing for their resolution and maximization of the organization's potential (Michalski and Kazienko, 2014).

ONA is an application of SNA in organizational contexts (Scott and Carrington, 2011; Freeman, 2012; Yang et al., 2016; Borgatti et al., 2018; SNA). Specifically, it refers to the study of intra-organizational networks (for more information on other applications of SNA in organizational contexts, see Yang et al., 2016) that, as in SNA, are defined by different types of relationships (ties or links) among the members (nodes or actors) of an organization (individuals or organizational units). Beginning with SNA's methods and instruments (see Scott and Carrington, 2011 and Borgatti et al., 2018 for a description of the main SNA indices and measures), in fact, ONA allows to comprehend, visualize, and monitor the interactions and relationships among organizational members or units (Garcia, 2015) that are crucial for elucidating organizational functioning: ≪*the organizational network is understood as a system of connections between people or organizational units (e.g., departments), created in order to exchange information, knowledge, ideas, and resources*≫ (Ujwary-Gil, 2020, p. 37).

To use ONA, organizations must first determine the professional families they are interested in and the type of analysis they wish to conduct (Hatala, 2006). Information about organizational network characteristics and employees can be gathered in a variety of ways (e.g., surveys, interviews, observations, emails, and organizational documents) (Garcia, 2015) and can concern not only the measurement of relationships/interactions among the nodes but also the analysis of some members' attributes that are useful to the organization in achieving its goals (e.g., personality traits for selection processes) (Wasserman and Faust, 1994; Scott, 2000; Hatala, 2006). Typically, surveys are the most common method for implementing ONA (Cross et al., 2002). Following the social network technique (Moreno, 1934), participants are provided with a list of all the names of the network members (or are asked to generate a list of specific members) against whom they have to indicate the nature of their relationships/interactions. In this context, the types of questions and the relationships to examine are of the utmost significance. In fact, for the consulting process to be successful, these questions must be relevant to the consulting objective (see Cross et al., 2002 for example questions). By collecting all this data, ONA enables examining node connections in an organizational network using graphs and matrices. Inter-organizational networks can be mathematically depicted as graphs (Borgatti et al., 2018). In addition, based on matrix algebra, the collected data can be used to calculate various indices and measures (Hatala, 2006; Müller-Prothmann, 2007; Borgatti et al., 2018), from which one can derive information on both inter-organizational interactions and the social positions held by the nodes of the network (see Newman, 2003; Scott and Carrington, 2011; Borgatti et al., 2018 for a description of the main indices). This information can be supplemented with data on the individual attributes of the nodes (typically detected via self-report questionnaires), facilitating in-depth knowledge of the organization's intangible assets. Consequently, through ONA, it is then possible to identify the critical elements (nodes/members/departments) that, within the network of connections, can facilitate the innovation of practices due to their social position and distinctive characteristics.

As a result, companies attempting to promote innovation can benefit from ONA data (Leenders and Dolfsma, 2016), which can be helpful in developing interventions aimed at facilitating communicative and relational exchanges among the various professional teams involved in delivering the service. Furthermore, the effectiveness of the entire service delivery process can be improved by identifying the specific actors essential for its promotion and support. For these reasons, ONA is a valuable organizational resource for developing and implementing innovative services.

## Methods integration

**Figure 2:**
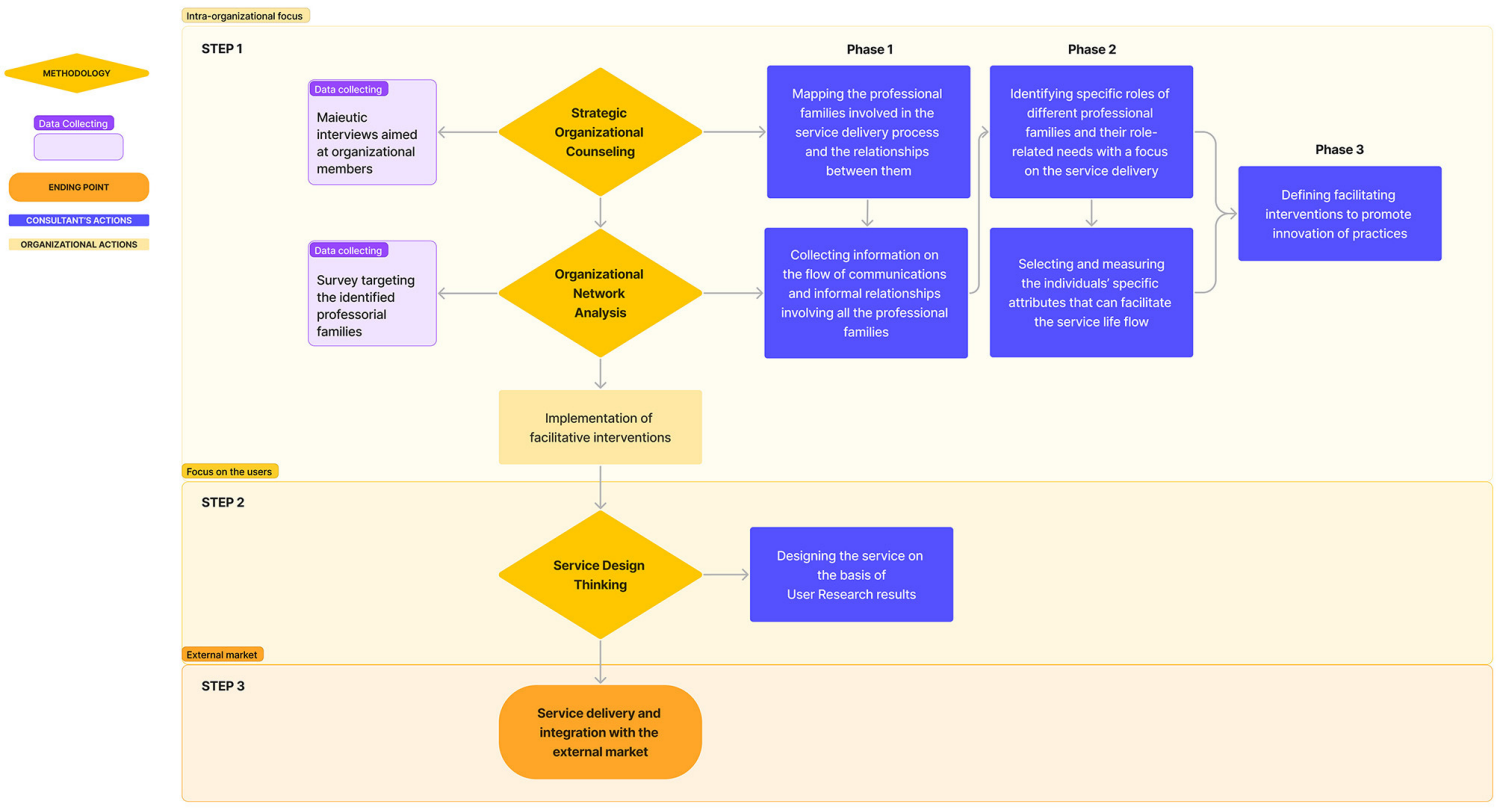
Service innovation process.

Based on the literature presented to date, we believe the integration between SOC and ONA provides a robust framework for conducting applied research and consultancy in the organizational field. The proposal for this integration within the service innovation process is illustrated in Figure 2. The methodologies and the specific actions described below can assist a company in successfully delivering its innovative service in the external market.

As described in Figure 2, the first step of the innovation process focuses on the intra-organizational processes. In fact, as mentioned earlier, SOC plays a pivotal role in paving the way for innovating organizational practices supporting new service delivery. More specifically, the implementation of SOC can be proposed as a strategic intervention to enhance the effectiveness and efficiency of the service delivery process, particularly in the case of complex organizations with multiple departments. SOC approach involves targeted maieutic interviews, both individual and group, with those engaged in the service's development. Through these interviews, different professional families involved in the service delivery process can be detected within the organization. During these sessions, it is possible to identify the specific role-related needs and challenges faced by each professional family, which may be supported for facilitating the service life flow. At this point, ONA can be implemented, involving all professional family members, to integrate SOC in its strategic intervention. Specifically, while mapping the professional families involved in the service delivery process and their relationships (SOC Phase 1), ONA can be used to collect information on informal exchanges among members of the professional families involved in the service delivery process (egocentric network) and simultaneously detect the characteristics of the organizational group as a whole (sociocentric network) (Forsyth, 2018). For instance, using various forms of data (Michalski and Kazienko, 2014), the connections between organizational members within and between professional families can be measured. This would make it possible, among other things, to highlight fewer evident relationships within the organizational chart, compare formal with informal structures, identify critical figures to facilitate decision-making or the flow of information and evaluate the level of group cohesion. Subsequently, during phase 2 of SOC, through an accurate analysis of the emerging *specific role-related needs*, ONA can be useful to select and measure the specific attributes of the individuals that can facilitate the service life flow. During this phase, the expert psychologist consultant chooses the psychological theoretical models to select the *Ambassadors* in the specific organizational environment. For instance, if the organization is interested in the leadership process, ONA can be used to identify the personality traits of each actor in the organizational network. This phase aims to identify the actors who, because of their social position and personal characteristics, can favorably influence the organization's performance, improving the organizational flow. In the final phase of Step 1 (see Figure 2), due to the integration of SOC and ONA, the organization will be able to create facilitation interventions to foster practice innovation based on the knowledge gathered in the previous phases. The advantage of using both of these methodologies is twofold. On one hand, ONA provides valuable information about the intra-organizational microsystem. On the other hand, SOC methodology offers a higher-level view of the organizational processes that underlie the development of innovative services.

Subsequently, Step 2 of Figure 2 involves using the Service Design Thinking^1^ methodology (Brown, 2009; Stickdorn and Schneider, 2011) in defining the service through a user-centered perspective. Indeed, by incorporating user feedback and insights throughout the design and development process, this approach ensures that the service effectively serves users by addressing their needs and represents the complementary aspect of innovation activities that are vital for the service's success. However, since this paper focuses on organizational processes, and given the extensive literature available on these topics, for further insights, please refer to Talamo et al. (2011) and Talamo et al. (2021).

In the end, after focusing on the intra-organizational processes necessary for implementing innovative practices and defining the service addressing the emerging users' needs, the service could be effectively delivered and launched in the external market (Step 3 in Figure 2).

The research and consulting activities outlined in the workflow do not conclude upon the service integration with the external market. Given the importance of quantifying innovation process efficiency and considering the high adaptability and flexibility required by organizations operating in constantly changing environments, we believe the workflow should be monitored cyclically. This monitoring should encompass not only the achievements' assessment (e.g., measurement of user feedback, customer involvement, customer satisfaction) but also the identification of new obstacles or challenges that may arise during service delivery in response to the complexity of the innovation process.

## Conclusions

Within the general framework of S*ectoral Innovation Systems* (Malerba, 2002), this paper presents a novel approach to enhancing service innovation within complex organizations, assuming both a micro and macro viewpoint. Specifically, we propose the integration of two research tools: Strategic Organizational Counseling (SOC; Talamo et al., 2021) and Organizational Network Analysis (ONA; Borgatti and Molina, 2003; Garcia, 2015) methodologies.

At macro level, SOC supports complex organizations in identifying and defining the professional families that need to be triggered for integrating the service with the external market. This methodology employs maieutic interviews and psychological techniques to uncover organizational challenges and specific role-related needs that may arise within the professional families during the service delivery process.

Moreover, SOC proposes facilitating interventions to overcome possible obstacles and satisfy organizational needs, with the final scope of supporting innovative practices. To address the micro-level within complex organizations, ONA is integrated. From this perspective, ONA emerges as a valuable resource in identifying people and processes that can assist the organization in promoting innovative practices that can facilitate the life flow of the service.

By integrating both approaches and combining qualitative and quantitative methods, researchers and consultants can leverage the strengths of each method and obtain a more comprehensive analysis of the organizational networks and processes for service innovation. In the end, through this comprehensive understanding, companies can effectively implement evidence-based practices and prioritize interventions which address specific organizational needs.

However, this paper exclusively presents research perspectives on the integration of these methodologies. This approach restricts the possibility to demonstrate the effectiveness of such integration and, simultaneously, to verify the emergence of any limitations and/or resistance within organizations. Therefore, a case study would be appropriate for future research to concretely illustrate how these methodologies are implemented in real organizational contexts.

## Data availability statement

The original contributions presented in the study are included in the article/supplementary material, further inquiries can be directed to the corresponding author.

## Author contributions

SM: Conceptualization, Writing—original draft, Writing—review and editing. MM: Conceptualization, Writing—original draft, Writing—review and editing. AT: Conceptualization, Supervision, Writing—review and editing.

## Footnotes

^1^For further insights on how the modeling of SOC can complement Service Design Thinking techniques, see Talamo et al. (2021).
